# 

**Published:** 1882-04

**Authors:** 


NOTICES.—In answer to numerous inquiries, we wish to state distinctly that W. H. Clark, the author of "Horses' Teeth," a revised and illustrated edition of which is now being prepared, has no connection whatever with R. E. Clark, who has traveled about the country as an itinerant veterinary surgeon.

Dr. John N. Navin's article on "The Expansion and Contraction of the Horse's Foot" will be concluded in the next number of the JOURNAL.

We are indebted to Captain Peter F. Alba, of Mobile, who is a leading authority in everything relating to horses, for papers and documents.

We desire to call the attention of our subscribers to whom bills are sent with this number, requesting that they will forward the amount either by postal order, registered letter, or draft on New York or other cities.

NOTICE OF ADVERTISEMENTS.—Especial attention is called to the Veterinary Surgeon's Visiting Chest, an advertisement of which appears in our present number. It is exceedingly convenient, cheap, and portable. With an extra dozen bottles it makes a very complete portable pharmacy. Those who have tested its merits practically consider it indispensable for all ordinary practice. The convenience of the small alcohol lamp which it contains is really worth the cost of the entire contents.

---

ACKNOWLEDGEMENT.—Thanks are due Prof. Robert Jennings, of Detroit, Mich., for a donation of some exceedingly well prepared specimens of bovine tuberculosis, also for some rare illustrations of "aged wolf teeth."

Two very large specimens of intestinal calculi have been received for the College Museum, the gift of J. W. Hawk, D.V.S., of Newark, N. J. Also a six months' fœtus and placenta of a sea-lion.

E. S. BATES,
*Dean Columbia Veterinary College.*

### BOOKS AND PAMPHLETS RECEIVED.

ELEMENTS DE THERAPEUTIQUE DOSIMETRIQUE ou restauration de la medecine Hippocratique avec les agents Therapeutiques de la science contemporaine. Par le Docteur FELIX PAQUET. Paris.

TRAITEMENT DOSIMETRIQUE DE LA DIPHTHERIE par le sulfure de calcium. Memoire communique par le Docteur P. A. FONTAINE au Congres International de Medecine Dosimetrique de Madrid.

THE BRAIN OF THE CAT. Preliminary account of the Gross anatomy. By BURT G. WILDER, M.D. pp. 40. pl. 4. 8vo. Read before the American Philosophical Society.

CURRENT FALLACIES ABOUT VACCINATION. A letter to Dr. W. B. Carpenter, C.B. By P. A. TAYLOR. M.P. Second edition. London: 1881. 8vo. pp. 37.

BY-LAWS OF THE NEW YORK COUNTY VETERINARY MEDICAL SOCIETY. Incorporated 1879. 8vo. pp. 11.

REPORT OF THE COMMISSION ON DOMESTIC ANIMALS. Made to the Connecticut Board of Agriculture. Hartford: 1882. 8vo. pp. 24.

FIFTH ANNUAL REPORT OF THE BOARD OF HEALTH of the State of New Jersey: 1881. Mount Holly, N. J. 8vo. pp. 344.

ANNUAL REPORT OF THE COMMISSIONER OF AGRICULTURE for the year 1880. 8vo. pp. 672. Plates. Washington: 1881.

STUDIES FROM THE BIOLOGICAL LABORATORY. Johns Hopkins University. Vol. II. No. 2.

THE VETERINARY JOURNAL. London.

WOCHENSCHRIFT FUR THIERHEILKUNDE UND VIEHZUCHT. Augsburg.

SCHWEIZERISCHES ARCHIV FUR THIERHEILKUNDE UND THIERZUCHT. Bern.

EL MEDICO Y CIRIYANO CENTRO-AMERICANO. Guatemala.

REPERTOIRE UNIVERSEL DE MEDECINE DOSIMETRIQUE. Paris.

THE CHICAGO MEDICAL JOURNAL AND EXAMINER. Chicago.
THE AMERICAN NATURALIST. Philadelphia.
NATIONAL LIVE STOCK JOURNAL. Chicago.
THE MEDICAL RECORD. New York.
THE BLACKSMITH AND WHEELWRIGHT. New York.
THE COLLEGE AND CLINICAL RECORD. Philadelphia.
ANNALS AND ANATOMY OF SURGERY. Brooklyn.
NEW ENGLAND MEDICAL MONTHLY. Newton, Conn.
THE CULTIVATOR AND COUNTRY GENTLEMAN. Albany.
INDIANA FARMER. Indianapolis.
MIRROR AND FARMER. Manchester, N. H.
CHICAGO MEDICAL REVIEW. Chicago.
ST. LOUIS CLINICAL RECORD. St. Louis.
JOURNAL OF NERVOUS AND MENTAL DISEASES. New York.
PEN AND PLOW. New York.
THE ROCKY MOUNTAIN MEDICAL TIMES: A monthly journal of medical, surgical and obstetrical science. Denver.
JOHNS HOPKINS UNIVERSITY CIRCULARS. Baltimore.
WALSH'S RETROSPECT. Washington.
VIRGINIA MEDICAL MONTHLY. Richmond.
THE WESTERN MEDICAL REPORTER. Chicago.
THE MEDICAL SUMMARY: A monthly journal devoted to practical medicine and new preparations. Lonsdale, Pa.

